# Supplementary material for: Wnt signaling in liver disease: emerging trends from a bibliometric perspective
Source: PeerJ. 2019 Jun 21;7:e7073. doi: 10.7717/peerj.7073 (PMC6590390; doi:10.7717/peerj.7073)
Supplement: Supplemental Information 2 [file peerj-07-7073-s002.pdf]

a. Visulization for all keywords ranged by their calculated values

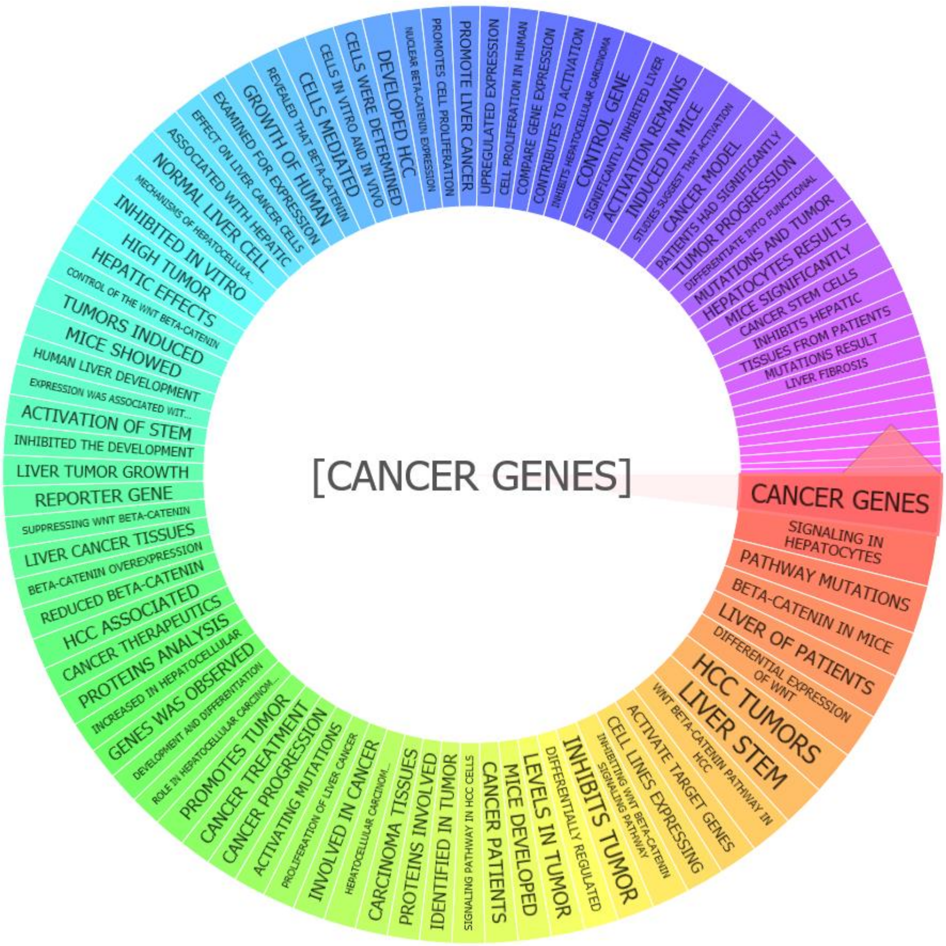

b. Top 10 keywords and their values

- Cancer Genes (357)
- Signaling in Hepatocytes (313)
- Pathway Mutations (290)
- Beta-catenin in Mice (276)
- Liver of Patients (276)
- Differential Expression of Wnt (273)
- HCC Tumors (267)
- Liver Stem (259)
- Wnt Beta-catenin Pathway in HCC (259)
- Activate Target Genes (236)
