# Supplementary material for: Wnt signaling in liver disease: emerging trends from a bibliometric perspective
Source: PeerJ. 2019 Jun 21;7:e7073. doi: 10.7717/peerj.7073 (PMC6590390; doi:10.7717/peerj.7073)
Supplement: Supplemental Information 3 [file peerj-07-7073-s003.pdf]

Top 43 Terms with the Strongest Citation Bursts

Terms	Year	Strength	Begin	End	1990 - 2018
beta-catenin gene	1990	8.3092	**1998**	2005	
signaling pathway	1990	2.2313	**2000**	2001	
nuclear accumulation	1990	2.9721	**2002**	2005	
adenomatous polyposis coli	1990	6.4555	**2002**	2006	
genetic alterations	1990	3.7144	**2002**	2006	
beta-catenin mutations	1990	9.2414	**2002**	2005	
binding site	1990	3.1096	**2004**	2007	
gene expression	1990	2.9711	**2004**	2009	
european association	1990	4.4468	**2005**	2006	
beta-catenin pathway	1990	6.687	**2005**	2006	
expression pattern	1990	3.6033	**2005**	2008	
hepatocellular carcinomas	1990	8.0349	**2007**	2011	
canonical wnt	1990	6.8028	**2007**	2011	
wnt pathway	1990	2.8812	**2008**	2011	
liver development	1990	6.2437	**2008**	2009	
progenitor cells	1990	8.8815	**2009**	2010	
hepatic stellate cells	1990	1.9561	**2009**	2010	
stem cells	1990	5.667	**2009**	2011	
hepatic progenitor cells	1990	4.8503	**2010**	2011	
recent study	1990	7.2698	**2010**	2013	
aberrant activation	1990	3.9739	**2010**	2011	
tumor growth	1990	1.2367	**2011**	2012	
hcc cell lines	1990	2.2286	**2012**	2014	
important role	1990	3.8018	**2012**	2013	
hepatitis b virus	1990	6.5583	**2012**	2014	
cyclin d1	1990	1.0341	**2012**	2013	
mouse model	1990	2.5254	**2013**	2015	
dose-dependent manner	1990	5.9011	**2013**	2014	
beta-catenin expression	1990	2.1505	**2013**	2014	
transcription factor	1990	4.4366	**2014**	2015	
overall survival	1990	5.7983	**2014**	2016	
hepg2 cells	1990	5.9518	**2014**	2018	
beta-catenin activation	1990	1.2928	**2014**	2015	
liver regeneration	1990	0.538	**2015**	2016	
critical role	1990	1.2637	**2015**	2016	
human hepatocellular carcinoma	1990	4.8092	**2015**	2016	
cell lines	1990	6.7347	**2015**	2018	
liver metastasis	1990	5.9868	**2015**	2016	
liver fibrosis	1990	6.8307	**2015**	2016	
hcc tissues	1990	6.4342	**2016**	2018	
cell migration	1990	5.6146	**2016**	2018	
hcc cells	1990	1.1262	**2016**	2018	
epithelial-mesenchymal transition	1990	6.939	**2016**	2018	
